# Pre-implantation and polar body diagnosis in cases of parental chromosomal translocations applying array-CGH

**DOI:** 10.1186/1755-8166-7-S1-I49

**Published:** 2014-01-21

**Authors:** Rolf-Dieter Wegner, Markus Stumm, Heide Mundt, Matthias Bloechle

**Affiliations:** 1 Zentrum für Pränataldiagnostik und Humangenetik – Kudamm 199, Berlin, Germany; 2 BG Berlin Genetics GmbH, Berlin, Germany; 3 Freie Universität Berlin, Germany; 4 Kinderwunschzentrum an der Gedächtniskirche, Berlin, Germany

## 

Parents with a balanced chromosomal translocation show an increased risk of reduced fertility including spontaneous abortions and chromosomally unbalanced offspring. In the course of genetic counseling the parents frequently decide to seek artificial reproductive techniques. In vitro fertilization (IVF) offers the chance to perform Polar Body Diagnosis (PBD) or Preimplantation Diagnosis (PID). Female translocation carriers can opt for analysis of polar body, blastomeres or trophectoderm cells while male translocation carriers have only a choice between blastomere and trophectoderm diagnostic.

In Germany, up to the year 2010 a restrictive “Embryo Protection Law” did allow only PBD excluding male translocation carriers from diagnostics. Thus, experience with PBD applying FISH or array-CGH was collected in cases of maternal translocations. For such cases, advantages and limits of array analysis as compared to the FISH approach will be presented. Furthermore, the resolution power of the BlueGnome 24sure and 24sure+ arrays will be shown. In particular we report here on the segregation pattern of maternal translocation chromosomes in 16 cases including 24 cycles and analyzing 97 first polar bodies. In addition to the distribution of the translocation chromosomes the number of segregation errors leading to aneuploidy in the 1^st^. polar body will be presented.

Since a German high court ruled that PID of trophectoderm cells should be legal under very stringent conditions, the Embryo Protection Law had been slightly modified in 2011. In future this will allow the application of array analysis also in cases of paternal translocation.

